# Supplementary material for: Arf6 regulates the cycling and the readily releasable pool of synaptic vesicles at hippocampal synapse
Source: eLife. 2016 Jan 5;5:e10116. doi: 10.7554/eLife.10116 (PMC4764570; doi:10.7554/eLife.10116)
Supplement: Supplementary file 1. — DOI: http://dx.doi.org/10.7554/eLife.10116.017 [file elife-10116-supp1.docx]

Erica Tagliatti, Manuela Fadda, Antonio Falace, Fabio Benfenati, Anna Fassio

***Arf6 regulates the cycling and the readily releasable pool of synaptic vesicles at hippocampal synapses***

**Table 1**: Synaptic area and AZ length in the various experimental groups.

| Group | Treatment | N | Synaptic area (µm^2^) | AZ length (µm) |
| --- | --- | --- | --- | --- |
| Control | - | 214 | 0.87±0.02 | 0.45±0.02 |
|  | 12 h TTX | 80 | 0.88±0.02 | 0.47±0.03 |
|  | 24 hTTX | 80 | 0.85±0.05 | 0.50±0.02 |
| Arf6-KD | - | 242 | 0.89±0.02 | 0.42±0.01 |
|  | 12 h TTX | 80 | 0.86±0.07 | 0.45±0.05 |
|  | 24 hTTX | 80 | 0.87±0.07 | 0.46±0.08 |
|  | cherry | 100 | 0.84±0.03 | 0.41±0.01 |
|  | Arf6Res-cherry | 100 | 0.85±0.02 | 0.42±0.02 |
| Not transduced | 3 h Vehicle | 75 | 0.85±0.02 | 0.40±0.01 |
|  | 72 h Vehicle | 80 | 0.89±0.01 | 0.44±0.04 |
|  | 3 h SecinH3 | 75 | 0.84±0.02 | 0.41±0.02 |
|  | 72 h SecinH3 | 88 | 0.94±0.07 | 0.41±0.01 |

Morphometric analysis of synaptic area and AZ length calculated for the various experimental groups. N is number of synapses counted for the listed treatment in the three experimental groups.
